# Supplementary material for: The Fission Yeast RNA-Binding Protein Meu5 Is Involved in Outer Forespore Membrane Breakdown during Spore Formation
Source: J Fungi (Basel). 2020 Nov 13;6(4):284. doi: 10.3390/jof6040284 (PMC7712723; doi:10.3390/jof6040284)
Supplement: Supplementary file 1 [file jof-06-00284-s001.zip › FigureS.pdf]

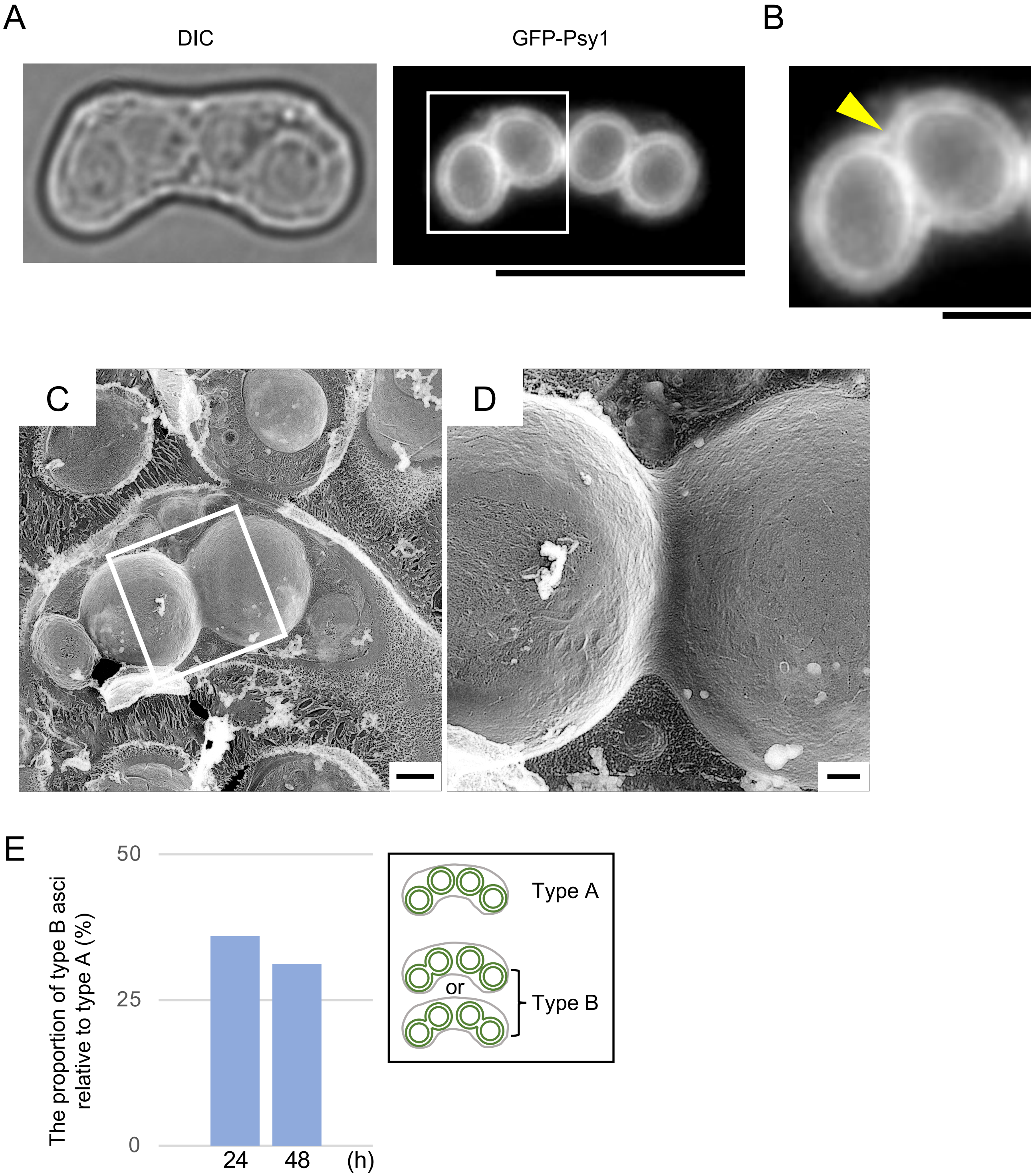

Figure S1. The outer FSM fuses with a neighboring spore in the *meu5-E22* mutant. *meu5-E22* (E22) cells were sporulated on ME medium for 2 days and analyzed by fluorescence microscopy (**A**,**B**) and quick-freeze deep-etch replica electron microscopy (**C**,**D**). High-magnification images of the region in the white square are shown on the right. Yellow arrowhead indicates the possible outer layer of the FSM. Bar, 10 μm (**A**); 2 μm (**B**); 1 μm (**C**); 0.3 μm (**D**). (**E**) Outer FSM fusion frequency in the *meu5-E22* mutant. Cells were cultured on SSA medium for 24 or 48 hours and analyzed by fluorescence microscopy (n>200). The proportion of type B asci relative to type A asci was quantified.

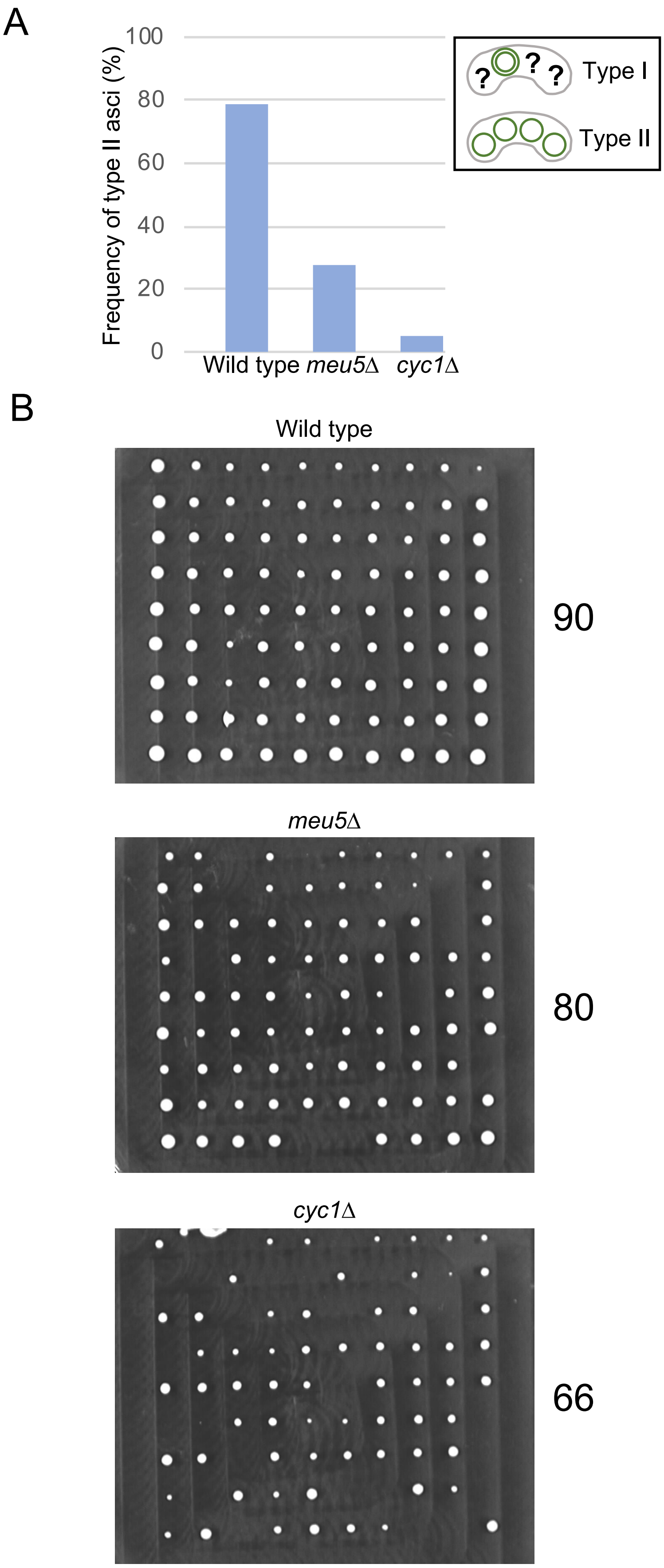

Figure S2. Ascus germination rates of *meu5Δ* and *cyc1Δ*. Wild-type (KI36), *meu5Δ* (BW85), and *cyc1Δ* (BW22) cells were sporulated on SSA medium for 2 days. (**A**) Frequency of outer FSM breakdown. (**B**) Germination of *meu5Δ* and *cyc1Δ* spores. For each strain, 90 asci were placed on YE medium and incubated at 30˚C for 4 days. The number of colonies was counted.
