# Supplementary material for: The Fission Yeast RNA-Binding Protein Meu5 Is Involved in Outer Forespore Membrane Breakdown during Spore Formation
Source: J Fungi (Basel). 2020 Nov 13;6(4):284. doi: 10.3390/jof6040284 (PMC7712723; doi:10.3390/jof6040284)
Supplement: Supplementary file 1 [file jof-06-00284-s001.zip › TableS1.pdf]

Table S1. Strain list

Strain	Genotype	Source
ET1 (FY25629)	h^{90} *ade6<<GFP-psy1 ura4-D18*	This study
E22 (FY39280)	h^{90} *ade6<<GFP-psy1 meu5-E22 ura4-D18*	This study
BW13 (FY39293)	h^{90} ade6*<<GFP-psy1 meu7::kanMX4 ura4-D18*	This study
BW15 (FY39295)	h^{90} ade6*<<GFP-psy1 meu30::kanMX4 ura4-D18 leu1-32*	This study
BW20 (FY39300)	h^{90} *leu1<<GFP-psy1 atg24::kanMX4 ade6-M216*	This study
BW22 (FY39302)	h^{90} *leu1<<GFP-psy1 cyc1::kanMX4 ade6-M216*	This study
BW23 (FY39303)	h^{90} *leu1<<GFP-psy1 mpf1::kanMX4 ade6-M216*	This study
BW24 (FY39304)	h^{90} *leu1<<GFP-psy1 uvi15::kanMX4 ade6-M210*	This study
BW26 (FY39306)	h^{90} *leu1<<GFP-psy1 hhp1::kanMX4 ade6-M210*	This study
BW28 (FY39308)	h^{90} *leu1<<GFP-psy1 pso2::kanMX4 ade6-M210*	This study
BW29 (FY39309)	h^{90} *leu1<<GFP-psy1 SPAC977.06::kanMX4 ade6-M216*	This study
BW40 (FY39320)	h^{90} *leu1<<mCherry-psy1 isp3-GFP*	This study
BW41 (FY39321)	h^{90} *ade6<<GFP-psy1 meu5::kanMX4 ura4-D18*	This study
BW46 (FY39326)	h^{90} *ade6<<GFP-psy1 meu5::meu5-3HA-kanMX6 ura4-D18*	This study
BW47 (FY39327)	h^{90} *ade6<<GFP-psy1 meu5-E22::meu5-E22-3HA-kanMX6 ura4-D18*	This study
BW84 (FY39364)	h^{90} *leu1<<GFP-psy1 meu5::kanMX4*	This study
BW85 (FY39365)	h^{90} *leu1<<GFP-psy1 meu5::KanMX4 ade6-M210*	This study
BW144 (FY39424)	h^{90} *ade6<<GFP-psy1 isp3::ura4 ura4-D18*	This study
BW162 (FY39442)	h^{90} *ade6<<mCherry-atb2 leu1<<GFP-psy1 meu5::kanMX4*	This study
BW303 (FY39583)	h^{90} *leu1<<mCherry-psy1 isp3-GFP cyc1::kanMX4*	This study
KI36 (FY25759)	h^{90} *leu1<<GFP-psy1 ade6-M210*	[30]
KI173 (FY25662)	h^{90} *ade6<<mCherry-atb2 leu1<<GFP-psy1*	[30]
STA71	h^{90} *leu1<<mCherry-psy1 isp3-GFP meu5::kanMX4 ade6-M216 ura4-D18*	Lab stock
YN68 (FY12296)	h^{90} *leu1<<GFP-psy1*	[48]
MU m58 (FY39640)	h^{90} *mug58::kanMX6 ade6<<GFP-psy1 his7::lacI-GFP lys1::lacO ura4-D18*	This study
MU m63 (FY39644)	h^{90} *mug63::kanMX6 ade6<<GFP-psy1 his7::lacI-GFP lys1::lacO ura4-D18*	This study
MU m67 (FY39648)	h^{90} *mug67::kanMX6 ade6<<GFP-psy1 his7::lacI-GFP lys1::lacO ura4-D18*	This study
MU m75 (FY39655)	h^{90} *mug75::kanMX6 ade6<<GFP-psy1 his7::lacI-GFP lys1::lacO ura4-D18*	This study
MU m86 (FY39665)	h^{90} *mug86::kanMX6 ade6<<GFP-psy1 his7::lacI-GFP lys1::lacO ura4-D18*	This study
MU m109 (FY39681)	h^{90} *mug109::kanMX6 ade6<<GFP-psy1 his7::lacI-GFP lys1::lacO ura4-D18*	This study
MU m110 (FY39682)	h^{90} *mug110::kanMX6 ade6<<GFP-psy1 his7::lacI-GFP lys1::lacO ura4-D18*	This study
MU m111 (FY39683)	h^{90} *mug111::kanMX6 ade6<<GFP-psy1 his7::lacI-GFP lys1::lacO ura4-D18*	This study
MU m113 (FY39685)	h^{90} *mug113::kanMX6 ade6<<GFP-psy1 his7::lacI-GFP lys1::lacO ura4-D18*	This study
MU m120 (FY39690)	h^{90} *mug120::kanMX6 ade6<<GFP-psy1 his7::lacI-GFP lys1::lacO ura4-D18*	This study
MU m129 (FY39698)	h^{90} *mug129::kanMX6 ade6<<GFP-psy1 his7::lacI-GFP lys1::lacO ura4-D18*	This study
MU m139 (FY39707)	h^{90} *mug139::kanMX6 ade6<<GFP-psy1 his7::lacI-GFP lys1::lacO ura4-D18*	This study
MU m142 (FY39710)	h^{90} *mug142::kanMX6 ade6<<GFP-psy1 his7::lacI-GFP lys1::lacO ura4-D18*	This study
MU m145 (FY39713)	h^{90} *mug145::kanMX6 ade6<<GFP-psy1 his7::lacI-GFP lys1::lacO ura4-D18*	This study
*are2*Δ	h^{90} *his5::URA3::his5 leu1<<GFP-psy1 sid4-tdTomato::hphMX6 ura4-D18 leu1-32* cyh^R *are2::kanMX4*	[49]
*pmp3*Δ	h^{90} *his5::URA3::his5 leu1<<GFP-psy1 sid4-tdTomato::hphMX6 ura4-D18 leu1-32* cyh^R *pmp3::kanMX4*	[49]
*SPBPB2B2.07c*Δ	h^{90} *his5::URA3::his5 leu1<<GFP-psy1 sid4-tdTomato::hphMX6 ura4-D18 leu1-32* cyh^R *SPBPB2B2.07c::kanMX4*	[49]
*agn2*Δ	h^{90} *his5::URA3::his5 leu1<<GFP-psy1 sid4-tdTomato::hphMX6 ura4-D18 leu1-32* cyh^R *agn2::kanMX4*	[49]
*ctl1*Δ	h^{90} *his5::URA3::his5 leu1<<GFP-psy1 sid4-tdTomato::hphMX6 ura4-D18 leu1-32* cyh^R *ctl1::kanMX4*	[49]
*pmc1*Δ	h^{90} *his5::URA3::his5 leu1<<GFP-psy1 sid4-tdTomato::hphMX6 ura4-D18 leu1-32* cyh^R *pmc1::kanMX4*	[49]
*fzr2*Δ	h^{90} *his5::URA3::his5 leu1<<GFP-psy1 sid4-tdTomato::hphMX6 ura4-D18 leu1-32* cyh^R *fzr2::kanMX4*	[49]
*ubp4*Δ	h^{90} *his5::URA3::his5 leu1<<GFP-psy1 sid4-tdTomato::hphMX6 ura4-D18 leu1-32* cyh^R *ubp4::kanMX4*	[49]
*fis1*Δ	h^{90} *his5::URA3::his5 leu1<<GFP-psy1 sid4-tdTomato::hphMX6 ura4-D18 leu1-32* cyh^R *fis1::kanMX4*	[49]

dsk1Δ	h^{90} *his5::URA3::his5 leu1<<GFP-psy1 sid4-tdTomato::hphMX6 ura4-D18 leu1-32 cyh*^{R} *dsk1::kanMX4*	[49]
SPAC750.06cΔ	h^{90} *his5::URA3::his5 leu1<<GFP-psy1 sid4-tdTomato::hphMX6 ura4-D18 leu1-32 cyh*^{R} *SPAC750.06c::kanMX4*	[49]
pcy1Δ	h^{90} *his5::URA3::his5 leu1<<GFP-psy1 sid4-tdTomato::hphMX6 ura4-D18 leu1-32 cyh*^{R} *pcy1::kanMX4*	[49]
meu6Δ	h^{90} *his5::URA3::his5 leu1<<GFP-psy1 sid4-tdTomato::hphMX6 ura4-D18 leu1-32 cyh*^{R} *meu6::kanMX4*	[49]
omh4Δ	h^{90} *his5::URA3::his5 leu1<<GFP-psy1 sid4-tdTomato::hphMX6 ura4-D18 leu1-32 cyh*^{R} *omh4::kanMX4*	[49]
gsk31Δ	h^{90} *his5::URA3::his5 leu1<<GFP-psy1 sid4-tdTomato::hphMX6 ura4-D18 leu1-32 cyh*^{R} *gsk31::kanMX4*	[49]
gor1Δ	h^{90} *his5::URA3::his5 leu1<<GFP-psy1 sid4-tdTomato::hphMX6 ura4-D18 leu1-32 cyh*^{R} *gor1::kanMX4*	[49]
SPBC1348.01Δ	h^{90} *his5::URA3::his5 leu1<<GFP-psy1 sid4-tdTomato::hphMX6 ura4-D18 leu1-32 cyh*^{R} *SPBC1348.01::kanMX4*	[49]
psk1Δ	h^{90} *his5::URA3::his5 leu1<<GFP-psy1 sid4-tdTomato::hphMX6 ura4-D18 leu1-32 cyh*^{R} *psk1::kanMX4*	[49]
elo1Δ	h^{90} *his5::URA3::his5 leu1<<GFP-psy1 sid4-tdTomato::hphMX6 ura4-D18 leu1-32 cyh*^{R} *elo1::kanMX4*	[49]
idn1Δ	h^{90} *his5::URA3::his5 leu1<<GFP-psy1 sid4-tdTomato::hphMX6 ura4-D18 leu1-32 cyh*^{R} *idn1::kanMX4*	[49]
ryh1Δ	h^{90} *his5::URA3::his5 leu1<<GFP-psy1 sid4-tdTomato::hphMX6 ura4-D18 leu1-32 cyh*^{R} *ryh1::kanMX4*	[49]
gma12Δ	h^{90} *his5::URA3::his5 leu1<<GFP-psy1 sid4-tdTomato::hphMX6 ura4-D18 leu1-32 cyh*^{R} *gma12::kanMX4*	[49]
git5Δ	h^{90} *his5::URA3::his5 leu1<<GFP-psy1 sid4-tdTomato::hphMX6 ura4-D18 leu1-32 cyh*^{R} *git5::kanMX4*	[49]
SPAC212.04cΔ	h^{90} *his5::URA3::his5 leu1<<GFP-psy1 sid4-tdTomato::hphMX6 ura4-D18 leu1-32 cyh*^{R} *SPAC212.04c::kanMX4*	[49]
SPAC212.01cΔ	h^{90} *his5::URA3::his5 leu1<<GFP-psy1 sid4-tdTomato::hphMX6 ura4-D18 leu1-32 cyh*^{R} *SPAC212.01c::kanMX4*	[49]
meu31Δ	h^{90} *his5::URA3::his5 leu1<<GFP-psy1 sid4-tdTomato::hphMX6 ura4-D18 leu1-32 cyh*^{R} *meu31::kanMX4*	[49]
pet2Δ	h^{90} *his5::URA3::his5 leu1<<GFP-psy1 sid4-tdTomato::hphMX6 ura4-D18 leu1-32 cyh*^{R} *pet2::kanMX4*	[49]
fbp1Δ	h^{90} *his5::URA3::his5 leu1<<GFP-psy1 sid4-tdTomato::hphMX6 ura4-D18 leu1-32 cyh*^{R} *fbp1::kanMX4*	[49]
SPAC4F10.16cΔ	h^{90} *his5::URA3::his5 leu1<<GFP-psy1 sid4-tdTomato::hphMX6 ura4-D18 leu1-32 cyh*^{R} *SPAC4F10.16c::kanMX4*	[49]
rit1Δ	h^{90} *his5::URA3::his5 leu1<<GFP-psy1 sid4-tdTomato::hphMX6 ura4-D18 leu1-32 cyh*^{R} *rit1::kanMX4*	[49]
gld1Δ	h^{90} *his5::URA3::his5 leu1<<GFP-psy1 sid4-tdTomato::hphMX6 ura4-D18 leu1-32 cyh*^{R} *gld1::kanMX4*	[49]
SPCC1739.08cΔ	h^{90} *his5::URA3::his5 leu1<<GFP-psy1 sid4-tdTomato::hphMX6 ura4-D18 leu1-32 cyh*^{R} *SPCC1739.08c::kanMX4*	[49]
pdh1Δ	h^{90} *his5::URA3::his5 leu1<<GFP-psy1 sid4-tdTomato::hphMX6 ura4-D18 leu1-32 cyh*^{R} *pdh1::kanMX4*	[49]
eng2Δ	h^{90} *his5::URA3::his5 leu1<<GFP-psy1 sid4-tdTomato::hphMX6 ura4-D18 leu1-32 cyh*^{R} *eng2::kanMX4*	[49]
mok14Δ	h^{90} *his5::URA3::his5 leu1<<GFP-psy1 sid4-tdTomato::hphMX6 ura4-D18 leu1-32 cyh*^{R} *mok14::kanMX4*	[49]
ndk1Δ	h^{90} *his5::URA3::his5 leu1<<GFP-psy1 sid4-tdTomato::hphMX6 ura4-D18 leu1-32 cyh*^{R} *ndk1::kanMX4*	[49]

KI36, KI173 and YN68 strains were provided by the National Bio-Resource Project (NBRP), Japan. The *S. pombe* strains constructed in this study have been deposited with the NBRP under the NBRP IDs shown here. Related strains are also deposited.
