# Supplementary material for: The Fission Yeast RNA-Binding Protein Meu5 Is Involved in Outer Forespore Membrane Breakdown during Spore Formation
Source: J Fungi (Basel). 2020 Nov 13;6(4):284. doi: 10.3390/jof6040284 (PMC7712723; doi:10.3390/jof6040284)
Supplement: Supplementary file 1 [file jof-06-00284-s001.zip › TableS2.pdf]

Table S2. DNA Primers

Name	Sequence
itr1f	5'-CTCTCTGTCCCCGGACAGTATGAAGATGAA-3'
itr1r	5'-GCTTTCAGCATTAACGAATTTGACGAACCC-3'
itr2f	5'-GGGTTCGTCAAATTCGTTAATGCTGAAAGC-3'
itr2r	5'-TCTGAGACTGTTTGAGTCCTGGCTTAACG-3'
meu5pc.1	5'-CACACGAGCTCCAGTGACAAAATCCGAGG-3'
meu5pc.2	5'-CACACGGGCCCGCAATAGCATGTGAGGCGG-3'
